# Patterns and socioeconomic differences in secondhand exposure to cigarettes, e-cigarettes, and heated tobacco products at home in Hong Kong adolescents

**DOI:** 10.18332/tid/186047

**Published:** 2024-05-15

**Authors:** Tianqi Chen, Man P. Wang, Yee Tak Derek Cheung, Lijun Wang, Tai Hing Lam, Sai Yin Ho

**Affiliations:** 1 School of Public Health, The University of Hong Kong, Hong Kong SAR, China; 2 School of Nursing, The University of Hong Kong, Hong Kong SAR, China

**Keywords:** secondhand tobacco exposure, secondhand exposure to e-cigarettes, socioeconomic differences, adolescents

## Abstract

**INTRODUCTION:**

Smoke or aerosols from cigarettes, e-cigarettes (ECs), or heated tobacco products (HTPs) are harmful. Yet, there is little knowledge about the specific patterns of secondhand tobacco exposure by source within household settings and the socioeconomic status (SES) differences in adolescents.

**METHODS:**

We used territory-representative student data from a cross-sectional school-based survey in 2020–2021 to calculate the weighted prevalence of secondhand exposure to cigarettes, e-cigarettes, and HTPs in the past seven days. Parental education and perceived family affluence were used as indicators of socioeconomic status. Generalized linear mixed models were used to analyze associations.

**RESULTS:**

Among 22039 participants, 29.8% reported any secondhand tobacco exposure (SH-Any) at home, primarily from cigarettes (27.4%), followed by e-cigarettes (4.0%) and HTPs (0.9%). Tertiary parental education level was associated with lower SH-Any exposure (Adjusted odds ratio, AOR=0.49; 95% CI: 0.45–0.53, p<0.001), fewer exposure days (β= -0.685, p<0.001), lower exposure to cigarettes (SH-CC) (AOR=0.49; 95% CI: 0.45–0.54, p<0.001) and to e-cigarettes or HTPs (SH-EC/HTP) (AOR=0.57; 95% CI: 0.45–0.71, p<0.001). 'Poor' family affluence was associated with higher exposures [AOR(SH-Any) =1.14; 95% CI: 1.06–1.22, p=0.001; β(days)=0.160, p<0.001; AOR(SH-CC) =1.15; 95% CI: 1.07–1.24, p<0.001], except for SH-EC/HTP exposure, which was higher in students in an affluent family (AOR =1.66; 95% CI: 1.25–2.21, p<0.001). Significant SES differences in SH-EC/HTP exposure were found only in groups with low parental education level. Dose-response relationships were found between lower SH-Any and SH-CC and higher SES categories (p for trend<0.001).

**CONCLUSIONS:**

Adolescents experienced a high prevalence of tobacco smoke exposure at home, primarily from cigarettes. Higher SES was associated with lower tobacco exposure, except for SH-EC/HTP, which was higher among adolescents from affluent families. Additionally, high parental education level was protective against exposure to SH-EC/HTP. Comprehensive control measures to reduce the use of these tobacco products are needed to protect adolescents of diverse socioeconomic backgrounds.

## INTRODUCTION

Secondhand smoke (SHS), defined as smoke from burning tobacco products (cigarettes, cigars, hookahs, and pipes)^1^, has been associated with diverse health outcomes in various authoritative reviews and reports in the early 21st century^2^. As the tobacco landscape evolved, electronic cigarettes (ECs) and heated tobacco products (HTPs) were introduced as alternatives to traditional cigarettes and gained popularity, raising scientific awareness of their exposure. Both ECs and HTPs are electronic heating systems – ECs produces vapors from mostly flavored e-liquids with nicotine, and HTPs heats tobacco sticks or ground leaves to approximately 300°C to generate tobacco smoke. Emissions from ECs and HTPs contain harmful substances such as formaldehyde, acrolein, heavy metals, phenolic compounds, and polycyclic aromatic hydrocarbons (PAHs), posing known and potential health risks^3,4^. The health effects of secondhand exposure to EC (SH-EC) and HTP (SH-HTP) on bystanders remain unclear because of the limited understanding of the exposure dose and the novelty of these products. Several cross-sectional studies in youth have associated the SH-EC aerosol with asthma exacerbations^5,6^ and the renormalization of tobacco smoking^7^.

Epidemiological data on secondhand EC (SH-EC) and HTP (SH-HTP) exposure in youth are limited. In US youth, 25.0% of middle and high school students were exposed to SH-EC in 2015, which increased to 33.2% in 2018^8^ and 44.4% in 2020, with 34.9% indoor exposure^9^. In Kuwait, 33.0% of high school students were exposed to SH-EC^6^. Research on SH-HTP exposure is mainly conducted in Japan, the most fertile market for HTPs, but results in youth have not been reported. In Japanese adults, exposure to SH-HTP increased from 4.5% in 2017 to 10.8% in 2020^10^.

Social disparities in youths’ exposure to SHS at home have been consistently documented, to be more likely in those living in socioeconomic disadvantage (e.g. with low parental education level^11^, low income^12^, low social class^13^, and poor neighbourhoods^14^). However, whether this applies to SH-EC remains unknown. We searched PubMed, EMBASE, Web of Science, and Google Scholar from 2000 to August 2023 using the keywords: ‘electronic cigarette/e-cigarette/EC exposure socioeconomic disparities/differences’ AND ‘heated tobacco product exposure socioeconomic disparities/differences’. Only one study in US youth reported sex and age differences in SH-EC exposure, with higher exposure in female and high school students^9^.

Hong Kong has continuously monitored the prevalence of smoking and tobacco smoke exposure in youth. Our previous study in Hong Kong adolescents found a high prevalence of SHS exposure from cigarettes at home, reaching 23.2% inside the home and 33.2% when including exposure from neighbours^15^. However, this study used data collected in 2010 when ECs and HTPs were not included. Hence, we aimed to thoroughly describe patterns of secondhand exposure to tobacco products inside the home by differentiating the product sources (cigarettes, ECs, and HTPs) and to examine how the overall secondhand exposure to any tobacco products (SH-Any exposure), SH-CC, SH-EC, and SH-HTP varies by socioeconomic status (SES) using territory-wide representative data.

## METHODS

### Study design

The school-based Survey on Smoking among Students (SSS) 2020–2021 is a biennial cross-sectional survey of Hong Kong secondary school students. The present survey was conducted from December 2020 to December 2021. Schools were selected from 18 districts in Hong Kong using a stratified random sampling method in proportion to the total number of schools in each district. All secondary 1–6 students (US grades 7–12) in selected schools, were invited to complete a paper-and-pencil questionnaire in the classroom administered by teachers and research staff or use an online version during school closure due to coronavirus disease 2019 (COVID-19). Informed consent was obtained from all parents for their children to participate before the survey. Students’ participation was voluntary, even with parental consent. Ethics approval was obtained from the Institutional Review Board (IRB) of the University of Hong Kong/Hospital Authority Hong Kong West Cluster (UW 20-569, approval date: 09/16/2020). The current study used data from 22039 students who participated in SSS 2020–2021 (n=25528) after excluding those with missing information on sex, age, grade, and tobacco smoke exposure at home.

### Demographics, SES indicators, and potential covariates

Information on sex (boys/girls) and age (years) was collected through the questionnaire. Harm perception towards secondhand exposure to tobacco products was measured using the question: ‘Do you think secondhand smoke of the following products (cigarettes, ECs, HTPs, water/hookah/shisha will harm your health’ with response options of ‘Definitely not’, ‘Probably not’, ‘Probably will’, and ‘Definitely will’. Students who answered ‘Definitely not’ or ‘Probably not’ to any of the mentioned products were classified as having uncertainty about harm perception regarding tobacco product exposure.

Parental education level and perceived family economic status were used as two indicators of SES. Parental education level was measured based on students’ answers to the question: ‘Among your parents, the highest level of education they received is: ‘Primary or below’, ‘Secondary’, ‘Post-secondary (e.g. university)’, ‘Don’t know’, and was classified into three groups ‘Secondary and below’, ‘Tertiary’, and ‘Don’t know’. Perceived family economic status was measured based on students’ answers to the question: ‘You consider your family's economic status as: ‘Relatively poor’, ‘Poor to average’, ‘Average’, ‘Average to rich’, or ‘Relatively rich’ and was classified into three groups: ‘Poor’, ‘Average’, or ‘Rich’.

A composite SES indicator was created by combining combining six levels of parental education (low/high) and perceived family affluence (poor/average/rich):

Low × Poor, Low × Average, Low × Rich, High × Poor, High × Average, and High × Rich. We chose the method ‘hot deck imputation’, which replaces missing cases on incomplete records (recipient) using values from complete observations of the same data set (donors) that matched the case that was missing^16^. Auxiliary variables used for matching donors to recipients included school ID, sex, age, and birthplace (Hong Kong/outside Hong Kong). Parental education level was categorized as ‘Low’ for ‘Secondary and below’ and ‘High’ for ‘Tertiary’.

Tobacco use status was measured using the question: ‘Please choose one option that suits you most regarding each of the following products (cigarette/electronic cigarette/heated tobacco product/waterpipe/other tobacco products, e.g. cigar and snus)’ with response options of ‘I have never used it’, ‘I have used it once or a few times (for fun or to try a puff)’, ‘I used to use it occasionally (not every day), but have quit now’, ‘I used to use it every day, but have quit now’, ‘I use it occasionally (not every day)’, and ‘I use it every day’. Students were classified as non-current tobacco users if they reported not currently using the mentioned tobacco products.

### Secondhand exposure to tobacco products inside the home

The specific tobacco sources of SHS exposure were measured based on students’ response of ‘yes’ to each option of the question: ‘In the past seven days, what smoking products have someone used near you at home?’ with options of ‘yes’ or ‘no’ to ‘Cigarette’, ‘Electronic cigarette’, ‘Heated tobacco product’, ‘Waterpipe/hookah/shisha’, ‘Other smoking products’ or ‘None of the above’. SH-Any exposure inside the home was defined as exposure to at least one tobacco product in the previous question and ≥1 day in the past seven days with the question: ‘On how many of the past seven days have someone used smoking products (including cigarette, electronic cigarette, heated tobacco product, etc.) near you at home?’ with response options from ‘0 days’ to ‘7 days’.

### Statistical analysis

All analyses were conducted using R (version 4.2.2). The weighted prevalence and mean exposure days of SH-Any exposure and the prevalence of secondhand exposure by specific sources (SH-CC, SH-EC, and SH-HTP) were calculated. Descriptive results were weighted by sex, age, and grade distribution of all Hong Kong students in the 2020–2021 school year, as provided by the Education Bureau of the Government of the Hong Kong SAR. The chi-squared test was used to examine differences in tobacco smoke exposure between sociodemographic groups.

Generalized linear mixed models (GLMM) with a ‘logit’ link function and linear mixed models (LMM) were used to explore the associations with SH-Any exposure and exposure days, respectively. Due to the limited sample size, SH-EC and SH-HTP were combined into SH-EC/HTP. Random intercepts were included to account for school clustering effects. Univariate analyses were initially done, and variables showing statistical significance were included in the multivariable models. As grade is highly correlated with age and the grade information was complete and reliable, we used grade instead of age to adjust together with sex, parental education level, family economic status, harm perception of SH-Any, tobacco use status, and school clustering effect. All these analyses were conducted using the package lme4 (version 1.1–21).

Significant effect modification was observed between parental education level and family economic status for SH-EC/HTP exposure, as well as between current tobacco use and the composite SES indicator for SH-Any and SH-EC/HTP (p<0.05). Sensitivity analyses were conducted using the composite SES indicator without imputation among non-current tobacco users. The models’ multiplicity was addressed by ensuring Variation Inflation Factor (VIF) values were <2. A two-sided p<0.05 was considered statistically significant. The sample size was calculated using G*Power software (version 3.1.9.6) with a power set to 0.95 and a type I error rate of 0.05 (two-tailed). For a small effect size of 0.10, the estimated sample size needed is 1545. Our sample of 22039 participants was much greater, and the post hoc power analysis for this sample size showed a >99% statistical power for a p<0.05.

## RESULTS

Among all 22039 students in this analysis, 51.2% were boys, 52.8% had parental education level as ‘Secondary and below,’ and 57.2% considered their family economic status as ‘Average’. The sample had a mean age (SD) of 15.1 (1.8) years. The unweighted characteristics of participants are shown in Supplementary file Table 1. As shown in Table 1, the weighted prevalence of SH-Any exposure in the past seven days was 29.8%, including 9.1% for 1–3 days, 4.5% for 4–6 days, and 16.1% for daily exposure. SH-Any was most prevalent in girls (32.4%), with parental education level as ‘Secondary and below’ (36.7%) and those from low-income families (36.0%) (p<0.001). Regarding tobacco sources, 27.4% of students reported exposure to SH-CC, 4.0% reported SH-EC, and 0.9% reported SH-HTP. SH-CC exposure was most prevalent in girls (30.1%), eldest students (28.2%), with parental education level as ‘Secondary and below’ (34.0%), and students from low-income families (33.8%) (p from <0.001 to 0.044). SH-EC and HTP exposure was also most prevalent in students with ‘Secondary and below’ parental education level (4.7% and 1.2%, respectively) (p all<0.001).

**Table 1:** Secondhand tobacco exposure at home in Hong Kong secondary school students by sociodemographic factors from a school-based cross-sectional study, 2020–2021 (N=22039)

*Characteristics*	*Weighted % (95% CI)*								*p ^a^* *p^SH-Any^* *p^SH-CC^* *p^SH-EC^* *p^SH-HTP^*
	*All*	*SH-Any*				*Tobacco exposure by product*			
		*≥1 days*	*1–3 days*	*4–6 days*	*Daily*	*SH-CC*	*SH-EC*	*SH-HTP*
**Overall**		29.8 (29.2–30.4)	9.1 (8.8–9.5)	4.5 (4.2–4.8)	16.1 (15.7–16.6)	27.4 (26.8–28.0)	4.0 (3.8–4.3)	0.9 (0.8–1.1)	
**Sex**									
Boys	51.2 (50.5–51.9)	27.2 (26.4–28.1)	8.6 (8.0–9.1)	4.1 (3.7–4.5)	14.6 (14.0–15.3)	24.8 (24.0–25.6)	3.5 (3.1–3.8)	1.0 (0.9–1.2)	<0.001 <0.001 <0.001 0.129
Girls	48.8 (48.1–49.5)	32.4 (31.5–33.3)	9.7 (9.2–10.3)	4.9 (4.5–5.4)	17.7 (17.0–18.5)	30.1 (29.3–31.0)	4.6 (4.2–5.0)	0.8 (0.7–1.0)	
**Age** (years), mean (SD)	15.1 (1.8)								
**Grade**									
S1–S2	26.4 (25.8–27.0)	29.1 (28.0–30.3)	9.6 (8.9–10.4)	4.3 (3.8–4.9)	15.1 (14.2–16.1)	26.3 (25.2–27.5)	4.5 (4.0–5.0)	0.9 (0.7–1.1)	0.107 0.044 0.050 0.753
S3–S4	36.0 (35.4–36.6)	29.4 (28.4–30.4)	9.5 (8.9–10.2)	4.5 (4.0–5.0)	15.4 (14.6–16.2)	27.4 (26.4–28.3)	3.6 (3.2–4.1)	0.9 (0.7–1.1)	
S5–S6	37.6 (37.0–38.3)	30.6 (29.6–31.6)	8.4 (7.8–9.0)	4.6 (4.2–5.1)	17.6 (16.8–18.4)	28.2 (27.3–29.2)	4.1 (3.7–4.6)	1.0 (0.8–1.2)	
**Parental education level**									
Secondary and lower	52.8 (52.2–53.5)	36.7 (35.8–37.6)	10.0 (9.5–10.6)	5.9 (5.5–6.3)	20.8 (20.1–21.6)	34.0 (33.2–34.9)	4.7 (4.3–5.1)	1.2 (1.0–1.4)	<0.001 <0.001 <0.001 <0.001
Tertiary	28.2 (27.6–28.8)	16.1 (15.2–17.1)	6.3 (5.7–6.9)	2.2 (1.9–2.6)	7.6 (7.0–8.3)	14.2 (13.4–15.1)	2.7 (2.3–3.1)	0.6 (0.4–0.8)	
Don’t know	19.0 (18.5–19.5)	30.3 (28.9–31.7)	10.6 (9.7–11.6)	4.2 (3.6–4.8)	15.5 (14.4–16.7)	28.2 (26.9–29.6)	4.2 (3.6–4.9)	0.7 (0.5–1.0)	
**Family economic status**									
Poor	30.9 (30.3–31.6)	36.0 (34.9–37.2)	10.4 (9.7–11.1)	5.0 (4.5–5.5)	20.7 (19.7–21.7)	33.8 (32.6–34.9)	3.8 (3.3–4.3)	1.2 (1.0–1.5)	<0.001 <0.001 0.075 0.008
Average	57.2 (56.6–57.9)	28.2 (27.4–29.0)	8.8 (8.3–9.3)	4.4 (4.1–4.8)	14.9 (14.3–15.6)	25.8 (25.1–26.6)	4.0 (3.7–4.4)	0.8 (0.6–0.9)	
Rich	11.8 (11.4–12.3)	21.3 (19.7–22.9)	7.6 (6.7–8.7)	3.7 (3.0–4.4)	10.0 (8.9–11.2)	18.5 (17.0–20.0)	4.8 (4.0–5.7)	1.1 (0.7–1.5)	

a Chi-squared tests.

As shown in Table 2, lower SES categories were associated with higher SH-Any exposure after adjusting for covariates (AOR_Tertiary education_=0.49; 95% CI: 0.45–0.53; AOR_Poor family_=1.18; 95% CI: 1.09–1.26) (p_Family income_ for trend<0.001). Moreover, greater exposure days were found in students with lower education level (adjusted β_Tertiary education_= -0.685, p<0.001, reference group= Secondary and lower) and those from poor families (adjusted β=0.160, p<0.001, reference group=Average). According to Table 3, lower SES was also associated with lower exposure to SH-CC (AOR_Tertiary education_=0.49; 95% CI: 0.45–0.54; AOR_Poor family_=1.15; 95% CI: 1.07–1.24) (p all<0.001). Two SES indicators had contrasting associations with SH-EC/HTP exposure: higher parental education level was associated with lower SH-EC/HTP exposure (AOR_Tertiary education_=0.57; 95% CI: 0.45–0.71), while a rich family was associated with higher exposure (AOR_Rich family_=1.66; 95% CI: 1.25–2.21) (p for trend<0.001). Significant interactions between parental education level and family economic status were found for SH-EC/HTP exposure and have been adjusted for in the model; in non-current tobacco users, similar associations were found between SES and tobacco exposure at home as in the total students (Supplemental file Table 2).

**Table 2:** Associations between socioeconomic factors and secondhand tobacco smoke exposure from any products at home in Hong Kong secondary school students from a school-based cross-sectional study, 2020–2021 (N=22039)

*Characteristics*	*SH-Any*		*Exposure days of SH-Any ^c^*	
	*OR ^a^ (95% CI)*	*AOR ^b^ (95% CI)*	*Estimate*	*SD*
**Sex**				
Boys ®	1	1		
Girls	1.27 (1.19–1.36)***	1.23 (1.15–1.31)***	0.177	0.036
**Grade**				
S1–S2 ®	1	1		
S3–S4	1.12 (1.04–1.21)**	1.06 (0.98–1.14)	0.048	0.041
S5–S6	1.02 (0.94–1.12)	0.90 (0.82–0.98)*	-0.045	0.047
p for trend	0.857	0.004^‡^	<0.001^‡^	
**Parental education level**				
Secondary ®	1	1		
Tertiary	0.48 (0.44–0.52)***	0.49 (0.45–0.53)***	-0.685***	0.044
Don’t know	0.72 (0.66–0.78)***	0.75 (0.69–0.81)***	-0.386***	0.045
**Family economic status**				
Poor	1.19 (1.11–1.28)***	1.14 (1.06–1.22)**	0.160***	0.039
Average ®	1	1		
Rich	0.83 (0.75–0.93)**	0.97 (0.87–1.08)	-0.038	0.053
p for trend	<0.001^†^	<0.001^†^	<0.001^†^	

a Adjusted for school clustering effect.

b AOR: adjusted odds ratio; adjusted for sex, age, grade, parental education level, family economic status, harm perception of tobacco smoke exposure, current tobacco use, and school clustering effect.

c Generalized mixed linear regression was conducted, adjusted for sex, age, grade, parental education level, family economic status, harm perception of tobacco smoke exposure, current tobacco use, and school clustering effect.

† Linear trend.

‡ Curvilinear trend.

® Reference categories.

* p<0.05,

** p< 0.01,

*** p<0.001.

**Table 3:** Secondhand exposure to cigarettes, ECs and HTPs at home in the past 7 days by socioeconomic characteristics from a school-based cross-sectional study, 2020-2021 (N=22039)

*Characteristics*	*SH-CC*		*SH-EC/HTP*	
	*OR ^a^ (95% CI)*	*AOR ^b^ (95% CI)*	*OR ^a^ (95% CI)*	*AOR ^b^ (95 % CI)*
**Sex**				
Boys ®	1	1	1	1
Girls	1.27 (1.19–1.36)***	1.23 (1.14–1.31)***	1.35 (1.18–1.55)***	1.34 (1.17–1.54)***
**Grade**				
S1–2 ®	1	1	1	1
S3–4	1.16 (1.07–1.25)***	1.10 (1.01–1.19)*	0.92 (0.79–1.07)	0.88 (0.75–1.03)
S5–6	1.07 (0.98–1.17)	0.94 (0.86–1.04)	0.76 (0.63–0.91)**	0.71 (0.59–0.86)***
p for trend	0.260	0.068	0.003^†^	<0.001^†^
**Parental education level**				
Secondary ®	1	1	1	1
Tertiary	0.47 (0.43–0.52)***	0.49 (0.45–0.54)***	0.64 (0.54–0.77)***	0.57 (0.45–0.71)***
Don’t know	0.72 (0.66–0.78)***	0.76 (0.70–0.82)***	0.82 (0.70–0.97)*	0.77 (0.62–0.95)*
**Family economic status**				
Poor	1.22 (1.13–1.30)***	1.15 (1.07–1.24)***	0.84 (0.72–0.98)*	0.76 (0.63–0.92)**
Average ®	1	1	1	1
Rich	0.79 (0.70–0.88)***	0.92 (0.82–1.03)	1.37 (1.14–1.65)**	1.66 (1.25–2.21)***
p for trend	<0.001^†^	<0.001^†^	0.093	0.037^†^

a Adjusted for school clustering effect.

b AOR: adjusted odds ratio; adjusted for sex, age, grade, parental education level, family economic status, harm perception of tobacco smoke exposure, current tobacco use, and school clustering effect.

† Linear trend.

‡ Curvilinear trend.

® Reference categories.

* p<0.05,

** p< 0.01,

*** p<0.001.

Table 4 shows the distribution of the composite SES and its association with tobacco exposure at home. Both crude and adjusted models found negative dose-response relations between SH-Any and SH-CC exposure at home (p for trend<0.001). Compared with the ‘Low × Poor’ group, the odds of SH-Any exposure decreased as the family economic status increased from poor to rich and parental education level increased from low to high (p all<0.05), except for the ‘Low × Rich’ group (AOR=0.88; 95% CI: 0.75–1.04) (p for trend<0.001). Odds of SH-CC exposure also decreased as the SES categories increased (AOR_Low_×_Average_=0.87; 95% CI: 0.80–0.94 and AOR_High_×_Rich_=0.46; 95% CI: 0.39–0.54) (p for trend<0.001). The ‘High × Rich’ group was at the lowest risk for SH-Any and SH-CC exposure at home. Regarding SH-EC/HTP, the ‘Low × Average’ (AOR=1.25; 95% CI: 1.06–1.48) and ‘Low × Rich’ (AOR=2.09; 95% CI: 1.51–2.90) group had 25% and 109% higher risk of exposure than the ‘Low × Poor’ group, respectively, while the differences in ‘High’ education groups are not statistically significant. Our results from the sensitivity analysis in non-current tobacco users were consistent (Supplementary file Table 3). Additionally, our sensitivity analysis using the composite SES indicator without imputation (Supplementary file Table 4) also aligns with the highest odds of SH-Any and SH-CC exposure found in the ‘Low × Poor’ group and the highest odds of SH-EC/HTP found in the ‘Low × Rich’ group (AOR=2.05; 95% CI: 1.48–2.82).

**Table 4:** Associations between secondhand tobacco smoke exposure at home and socioeconomic status (composite SES) in Hong Kong secondary school students from a school-based cross-sectional study, 2020–2021 (N=22039)

*Characteristics*	*% (95% CI)*	*SH-Any*		*SH-CC*		*SH-EC/HTP*	
		*OR ^a^ (95% CI)*	*AOR ^b^ (95% CI)*	*OR ^a^ (95% CI)*	*AOR ^b^ (95% CI)*	*OR ^a^ (95% CI)*	*AOR ^b^ (95% CI)*
**Composite SES**							
Low × Poor ®	25.5 (24.9–26.1)	1	1	1	1	1	1
Low × Average	35.7 (35.1–36.4)	0.91 (0.84–0.98)*	0.87 (0.81–0.95)**	0.89 (0.82–0.96)**	0.87 (0.80–0.94)**	1.34 (1.13–1.6)**	1.25 (1.06–1.48)**
Low × Rich	3.7 (3.4–3.9)	0.91 (0.77–1.07)	0.88 (0.75–1.04)	0.78 (0.66–0.92)**	0.78 (0.66–0.93)**	2.47 (1.88–3.25)***	2.09 (1.51–2.90)***
High × Poor	5.4 (5.1–5.7)	0.62 (0.53–0.72)***	0.58 (0.49–0.68)***^§^	0.59 (0.50–0.69)***	0.58 (0.49–0.68)***	1.01 (0.72–1.43)	0.76 (0.53–1.11)^§^
High × Average	21.7 (21.1–22.2)	0.53 (0.48–0.59)***	0.51 (0.46–0.56)***^§^	0.51 (0.46–0.57)***	0.50 (0.45–0.56)***	0.90 (0.72–1.11)	0.81 (0.54–1.20)
High × Rich	8.0 (7.7–8.4)	0.46 (0.40–0.54)***	0.45 (0.39–0.53)***	0.46 (0.39–0.53)***	0.46 (0.39–0.54)***	1.07 (0.80–1.43)	0.86 (0.51–1.46)^§^
p for trend		<0.001^†^	<0.001^†^	<0.001^†^	<0.001^†^	0.013^‡^	0.086

Low: parental education level as ‘Secondary and lower’. High: parental education level as ‘Tertiary’.

a Adjusted for school clustering effect.

b AOR: adjusted odds ratio; adjusted for sex, age, grade, parental education level, family economic status, harm perception of tobacco smoke exposure, current tobacco use, and school clustering effect.

† Linear trend.

‡ Curvilinear trend.

§ Interaction with current tobacco use.

® Reference categories.

* p<0.05,

** p< 0.01,

*** p<0.001.

## DISCUSSION

In this population-based study, we found that nearly one-third of adolescents were exposed to SH-Any in the home setting, with cigarettes being the primary product source. In addition, students with lower SES had higher SH-Any, SH-CC, and more exposure days at home, while increased family economic status was associated with higher SH-EC/HTP exposure. Negative dose-relations were found in SH-Any and SH-CC exposure, and SES categorizes were increased. To our knowledge, our study is the first to report the prevalence of secondhand exposure to tobacco by various products in youth and to explore socioeconomic differences in EC and HTP exposure in household settings.

In our results, 29.8% of adolescents were exposed to SH-Any inside the home, predominantly from cigarettes (27.4%), while exposure to ECs (4.0%) and HTPs (0.9%) remained at a relatively low level. These findings are consistent with the tobacco landscape in Hong Kong adults^17^, where ECs are less common than cigarettes. Previous research conducted at a global level and in other countries/regions has consistently reported that more than 30% of adolescents are exposed to SHS at home^9,18^. However, these studies either did not differentiate among tobacco products or solely encompassed exposure to cigarettes. Our study extends current knowledge by distinguishing among various sources of tobacco exposure. It facilitates cross-regional comparisons in areas sharing similar cultures and population densities, such as Singapore, Japan, and Korea.

Concerningly, the prevalence of SH-CC (27.4%) is disproportionally high compared with Hong Kong’s remarkably low cigarette smoking prevalence in those aged ≥15 years (9.5%), which is among the lowest in the world. The decline in smoking prevalence happened more than three decades ago, mainly due to large tax increases, and the recent rate of decline was small. Although recent publications in youth have reported a decreasing trend in SHS from cigarettes at home^19,20^, we observed an unexpected increase in SH-CC inside the home (27.4%) compared with our previous report (23.6%) using 2010–2011 data^15^. This finding could be partly attributed to an increased awareness of tobacco exposure due to health campaigns and underscores the need for smoking cessation efforts among adult smokers. Further investigation is warranted to elucidate the factors contributing to this change fully.

Our results on SES differences between SH-Any and SH-CC align with previous research on SHS exposure, which consistently links lower SES to increased exposure in youth^21,22^. This association could be attributed to a higher prevalence of residential smokers, more extended periods of exposure^23^, and a lower adoption rate of smoke-free homes^24^. We extend current knowledge by showing a dose-response relationship between reduced SH-Any, SH-CC exposure, and higher SES. Additionally, we found that a richer family was associated with increased SH-EC/HTP exposure, while higher parental education level served as a protective factor. The income-related difference in indoor EC/HTP exposure could be explained by the higher prevalence of ECs and HTP use among adults with higher incomes^25,26^, coupled with the perception that ECs and HTPs are less harmful and more acceptable for indoor use^27^. Although EC use is more prevalent in adults with higher education^28^, we found adolescents reporting ‘Tertiary’ parental education level were less exposed to SH-EC/HTP. One explanation, as has been reported previously, is that tobacco users in households with higher education level are more likely to adopt smoke-free bans at home^29^ and avoid exposing their children to tobacco smoke. A previous study in the UK supports this hypothesis by showing that people with high SES (characterized by occupation, which is highly correlated with education level) more strongly support the restriction of EC use in private settings^30^. Our study adds insights into the field of SH-EC and HTP exposure. It suggests that education may reduce SES disparities in adolescent family economic status, although future research from qualitative studies is needed to understand the complex interplay among SES factors.

Several countries and regions have implemented mandatory restrictions in private cars to protect children and adolescents from tobacco exposure. Still, comprehensive measures to restrict smoking in the home have not been proposed by any country, except for limited measures in the US related to government-funded housing and balcony smoking restrictions by housing associations and landlords^31^. These smoke-free bans on SHS have shown varying effectiveness in protecting children from exposure based on sex, age, country, and family SES^22,32^. Some countries, such as Greece, took a further step by introducing a ‘vape-free’ policy within private residences and vehicles^33^. In Hong Kong, the Marking Scheme for Estate Management Enforcement is the sole policy that regulates smoking in housing by allotting residents who smoke or carry a lighted cigarette in common areas^34^. After the present survey, Hong Kong banned the manufacture, import, advertising, distribution, and sale of ECs and HTPs since 30 April 2022; however, the use of these tobacco products remains legal, necessitating continued observation and evaluation of the impact of regulation. The current policies are not sufficient to fully protect Hong Kong youth from tobacco smoke exposure in private settings. Efforts such as legislation, education campaigns, and intervention research are necessary to ensure equitable protection for vulnerable groups.

### Strengths and limitations

One of the strengths of our study is that the SSS is a large, territory-representative survey. This provides a substantial sample size that allows for the analysis of associations with reasonable statistical power, ensuring the generalizability of the research findings to the broader adolescent population. Rigorous sensitivity analyses were conducted to reinforce the robustness of our results. Our study has limitations. First, self-reported tobacco exposure might be subject to recall biases and affected by individual awareness. However, objective measurements, such as salivary cotinine measures, are not feasible in the context of large-scale surveys. Furthermore, previous studies have also validated self-report measures against biomarkers of tobacco exposure^35^. Second, some students might have mistaken the source of tobacco products, particularly from relatively new products in Hong Kong, such as ECs and HTPs. To mitigate this potential issue, we briefly introduced cigarettes, ECs, and HTPs at the beginning of the questionnaire. Moreover, the possibility of residual confounding by unmeasured variables could not be excluded despite adjusting potential covariates related to tobacco smoke exposure when examining the related SES differences. The specific context of Hong Kong should be considered for the generalizability of our findings to other countries and regions. Nonetheless, our study could provide insights for areas with similar social contexts, especially those with low smoking prevalence and population density.

## CONCLUSIONS

We found a disproportionally high prevalence of secondhand exposure to tobacco products at home in adolescents given the low smoking prevalence among Hong Kong adults. Exposure to SH-Any and SH-CC decreased with higher SES in a dose-response pattern, whereas SH-EC/HTP exposure was positively associated with a family of high economic status. High parental education level had a protective effect against SH-EC/HTP exposure. Comprehensive and targeted tobacco control measures are necessary to improve health equity among diverse socioeconomic groups.

## Supplementary Material



## Data Availability

The data supporting this research are available from the authors on reasonable request.
