# Supplementary material for: Patterns and socioeconomic differences in secondhand exposure to cigarettes, e-cigarettes, and heated tobacco products at home in Hong Kong adolescents
Source: Tob Induc Dis. 2024 May 15;22:10.18332/tid/186047. doi: 10.18332/tid/186047 (PMC11095092; doi:10.18332/tid/186047)
Supplement: Supplementary file 1 [file TID-22-78-s1.pdf]

STROBE Statement—checklist of items that should be included in reports of observational studies

	Item No.	Recommendation	Page No.	Relevant text from manuscript
**Title and abstract**	1	(*a*) Indicate the study's design with a commonly used term in the title or the abstract	1	Cross-sectional Surveys
		(*b*) Provide in the abstract an informative and balanced summary of what was done and what was found	1	Abstract: Introduction
**Introduction**				
Background/rationale	2	Explain the scientific background and rationale for the investigation being reported	2	Introduction 1st -3rd paragraphs
Objectives	3	State specific objectives, including any prespecified hypotheses	2	Introduction 4th paragraph
**Methods**				
Study design	4	Present key elements of study design early in the paper	2	Methods: Study design
Setting	5	Describe the setting, locations, and relevant dates, including periods of recruitment, exposure, follow-up, and data collection	2	Methods: Study design
Participants	6	(*a*) *Cohort study*—Give the eligibility criteria, and the sources and methods of selection of participants. Describe methods of follow-up<br>*Case-control study*—Give the eligibility criteria, and the sources and methods of case ascertainment and control selection. Give the rationale for the choice of cases and controls<br>*Cross-sectional study*—Give the eligibility criteria, and the sources and methods of selection of participants	2	Methods: Study design
		(*b*) *Cohort study*—For matched studies, give matching criteria and number of exposed and unexposed		

		Case-control study—For matched studies, give matching criteria and the number of controls per case		
Variables	7	Clearly define all outcomes, exposures, predictors, potential confounders, and effect modifiers. Give diagnostic criteria, if applicable	3	Methods
Data sources/ measurement	8*	For each variable of interest, give sources of data and details of methods of assessment (measurement). Describe comparability of assessment methods if there is more than one group	3	Methods
Bias	9	Describe any efforts to address potential sources of bias	3-5	Methods: Statistical analysis
Study size	10	Explain how the study size was arrived at	2	Methods: Study design

Continued on next page

Quantitative variables	11	Explain how quantitative variables were handled in the analyses. If applicable, describe which groupings were chosen and why	3-5	Methods: Statistical analysis
Statistical methods	12	(*a*) Describe all statistical methods, including those used to control for confounding	3-5	Methods: Statistical analysis
		(*b*) Describe any methods used to examine subgroups and interactions	3-5	Methods: Statistical analysis
		(*c*) Explain how missing data were addressed		
		(*d*) *Cohort study*—If applicable, explain how loss to follow-up was addressed<br>*Case-control study*—If applicable, explain how matching of cases and controls was addressed<br>*Cross-sectional study*—If applicable, describe analytical methods taking account of sampling strategy	3-5	Methods: Statistical analysis
		(*e*) Describe any sensitivity analyses	3-5	Methods: Statistical analysis
**Results**				
Participants	13*	(a) Report numbers of individuals at each stage of study—eg numbers potentially eligible, examined for eligibility, confirmed eligible, included in the study, completing follow-up, and analysed	2	Methods: Study design
		(b) Give reasons for non-participation at each stage	2	Methods: Study design
		(c) Consider use of a flow diagram		
Descriptive data	14*	(a) Give characteristics of study participants (eg demographic, clinical, social) and information on exposures and potential confounders	5-6	Results 1st paragragh
		(b) Indicate number of participants with missing data for each variable of interest	2	Methods: Study design
		(c) *Cohort study*—Summarise follow-up time (eg, average and total amount)		
Outcome data	15*	*Cohort study*—Report numbers of outcome events or summary measures over time		
		Case-control study—Report numbers in each exposure category, or summary measures of exposure		

		Cross-sectional study—Report numbers of outcome events or summary measures	5-7	Results
Main results	16	(*a*) Give unadjusted estimates and, if applicable, confounder-adjusted estimates and their precision (eg, 95% confidence interval). Make clear which confounders were adjusted for and why they were included	Supplementary Table 1	Supplementary Table 1
		(*b*) Report category boundaries when continuous variables were categorized		
		(*c*) If relevant, consider translating estimates of relative risk into absolute risk for a meaningful time period		

Continued on next page

Other analyses	17	Report other analyses done—eg analyses of subgroups and interactions, and sensitivity analyses	Supplementary Table 2 to 4	Supplementary Table 2 to 4
**Discussion**				
Key results	18	Summarise key results with reference to study objectives	7	Discussion 1st paragraph
Limitations	19	Discuss limitations of the study, taking into account sources of potential bias or imprecision. Discuss both direction and magnitude of any potential bias	8-9	Discussion 8th paragraph
Interpretation	20	Give a cautious overall interpretation of results considering objectives, limitations, multiplicity of analyses, results from similar studies, and other relevant evidence	7-9	Discussion 2nd – 7th paragraph
Generalisability	21	Discuss the generalisability (external validity) of the study results	9	Discussion 8th paragraph
**Other information**				
Funding	22	Give the source of funding and the role of the funders for the present study and, if applicable, for the original study on which the present article is based	11	Funding

*Give information separately for cases and controls in case-control studies and, if applicable, for exposed and unexposed groups in cohort and cross-sectional studies.

Note: An Explanation and Elaboration article discusses each checklist item and gives methodological background and published examples of transparent reporting. The STROBE checklist is best used in conjunction with this article (freely available on the Web sites of PLoS Medicine at http://www.plosmedicine.org/, Annals of Internal Medicine at http://www.annals.org/, and Epidemiology at http://www.epidem.com/). Information on the STROBE Initiative is available at www.strobe-statement.org.

Supplementary Table 1. Characteristics of participants from a school-based cross-sectional study, 2020/2021 (N=22039)

Characteristics	Unweighted	
	N	%(95%CI) or mean(SD)
**Sex**		
Boys	12496	56.7(56.0-57.4)
Girls	9543	43.3(42.6-44.0)
**Mean age (SD)**	-	14.8(1.7)
**Grade**		
S1-S2	7129	32.3(31.7-33.0)
S3-S4	9019	40.9(40.3-41.6)
S5-S6	5891	26.7(26.1-27.3)
**Highest parental education**[a]		
Secondary and below	11204	51.0(50.3-51.7)
Tertiary	6349	28.9(28.3-29.5)
Don't know	4417	20.1(19.6-20.6)
**Family affluence**[a]		
Poor	6308	28.7(28.1-29.3)
Average	12887	58.6(58.0-59.3)
Rich	2778	12.6(12.2-13.1)
**Current tobacco use**[a]		
Cigarettes	296	1.3(1.2-1.5)

ECs	253	1.1(1.0-1.3)
Any tobacco products	394	4.4(4.1-4.7)
**Consider tobacco smoke exposure as harmful**		
Definitely yes	2082	9.4(9.1-9.8)
Uncertainty	19957	90.6(90.2-90.9)
**Tobacco smoke exposure inside the home**		
Any	6376	28.9(28.3-29.5)
Cigarettes	5876	26.7(26.1-27.2)
ECs	824	3.7(3.5-4.0)
HTPs	208	0.9(0.8-1.1)

[a] Frequency did not precisely correspond to prevalence due to missing values.

Supplementary Table 2. Associations between sociodemographic factors and secondhand tobacco smoke exposure at home in non-current tobacco users from a school-based cross-sectional study, 2020/2021 (n=21637)

**Overall**	SH-Any[a]	Exposure days of SH-Any[b]		SH-CC[a]	SH-EC/HTP[a]
	Adjusted OR[a](95%CI)	Estimate	SD	Adjusted OR[a](95%CI)	Adjusted OR[a](95%CI)
**Sex**					
Boys	reference	reference	/	reference	reference
Girls	1.22 (1.14-1.30)***	0.168***	0.036	1.22 (1.14-1.31)***	1.34 (1.16-1.55)***
**Grade**					
S1-2	reference	reference	/	reference	reference
S3-4	1.05 (0.97-1.13)	0.040	0.041	1.09 (1.00-1.18)*	0.89 (0.76-1.04)
S5-6	0.89 (0.81-0.98)*	-0.055	0.047	0.94 (0.85-1.03)	0.71 (0.59-0.87)**
P for trend	0.006[‡]	0.174	/	0.269	< 0.001[†]
**Highest parental education**					
Secondary	reference	reference	/	reference	reference
Tertiary	0.48 (0.44-0.53)***	-0.692***	0.044	0.48 (0.44-0.53)***	0.57 (0.45-0.72)***
Don't know	0.74 (0.68-0.80)***	-0.400***	0.045	0.75 (0.69-0.82)***	0.73 (0.58-0.91)**
**Family affluence**					
Poor	1.14 (1.06-1.23)***	0.168***	0.039	1.16 (1.08-1.25)***	0.76 (0.62-0.92)**
Average	reference	reference	/	reference	reference
Rich	0.96 (0.86-1.08)	-0.046***	0.053	0.92 (0.82-1.03)	1.65 (1.23-2.21)**
P for trend	< 0.001[†]	< 0.001[†]	/	< 0.001[†]	< 0.001[†]

[a] Adjusted for sex, age, grade, highest parental education, family affluence, harm perception regarding tobacco smoke exposure, current tobacco use and school clustering effect.

[b] Generalised mixed linear regression was conducted, adjusted for sex, age, grade, highest parental education, family affluence, harm perception of tobacco smoke exposure, current tobacco use and school clustering effect.

* $P < 0.05$, ** $P < 0.01$, *** $P < 0.001$.

† Linear trend; ‡ Curvilinear trend.

Supplementary Table 3. Associations between secondhand tobacco smoke exposure at home and socioeconomic status (composite SES) in non-current tobacco users from a school-based cross-sectional study, 2020/2021 (n=21637)

**Characteristics**	%(95%CI)	AOR (95% CI)					
		SH-Any		SH-CC		SH-EC/HTP	
		Crude OR[a] (95%CI)	Adjusted OR[b](95%CI)	Crude OR[a] (95%CI)	Adjusted OR[b](95%CI)	Crude OR[a] (95%CI)	Adjusted OR[b](95%CI)
**Composite SES**							
Low * Poor	25.4(24.8-25.9)	reference	reference	reference	reference	reference	reference
Low * Average	35.7(35.0-36.3)	0.9 (0.83-0.97)**	0.87 (0.81-0.95)**	0.88 (0.81-0.95)**	0.86 (0.79-0.93)***	1.33 (1.11-1.60)**	1.26 (1.05-1.52)*
Low * Rich	3.6(3.4-3.9)	0.87 (0.74-1.03)	0.88 (0.75-1.04)	0.77 (0.65-0.91)**	0.78 (0.66-0.93)**	2.35 (1.75-3.14)***	2.25 (1.68-3.01)***
High * Poor	5.4(5.1-5.7)	0.59 (0.50-0.7)***	0.58 (0.49-0.69)***	0.59 (0.50-0.69)***	0.58 (0.49-0.68)***	0.83 (0.56-1.22)	0.82 (0.55-1.21)
High * Average	21.9(21.3-22.4)	0.53 (0.48-0.58)***	0.51 (0.46-0.57)***	0.51 (0.46-0.56)***	0.50 (0.45-0.55)***	0.90 (0.72-1.13)	0.86 (0.68-1.08)
High * Rich	8.1(7.7-8.5)	0.45 (0.39-0.53)***	0.45 (0.39-0.53)***	0.45 (0.38-0.52)***	0.45 (0.38-0.53)***	0.98 (0.72-1.33)	0.93 (0.68-1.27)
P for trend		< 0.001[†]	< 0.001[†]	< 0.001[†]	< 0.001[†]	0.004[‡]	0.013[‡]

Low: Parental education as "Secondary and below"; High: Parental education as "Tertiary".

[a] adjusted for school clustering effect.

[b] adjusted for sex, age, grade, highest parental education, family affluence, harm perception regarding tobacco smoke exposure and school clustering effect.

* $P < 0.05$, ** $P < 0.01$, *** $P < 0.001$.

† Linear trend; ‡ Curvilinear trend.

Supplementary Table 4. Associations between secondhand tobacco smoke exposure at home and socioeconomic status (composite SES without imputation) in Hong Kong secondary school students from a school-based cross-sectional study, 2020/2021 (N=22039)

Characteristics	%(95%CI)	AOR (95% CI)					
		SH-Any		SH-CC		SH-EC/HTP	
		Crude OR[a] (95%CI)	Adjusted OR[b](95%CI)	Crude OR[a] (95%CI)	Adjusted OR[b](95%CI)	Crude OR[a] (95%CI)	Adjusted OR[b](95%CI)
**SES composite**							
Low * Poor	21.4(20.9-22.0)	reference	reference	reference	reference	reference	reference
Low * Average	28.3(27.7-28.9)	0.91 (0.84-1.00)*	0.87 (0.80-0.95)***¤	0.89 (0.81-0.97)**	0.85 (0.78-0.93)***	1.38 (1.15-1.67)**	1.31 (1.10-1.54)**
Low * Rich	3.2(3.0-3.4)	0.92 (0.78-1.10)	0.88 (0.74-1.06)	0.79 (0.66-0.95)*	0.79 (0.66-0.95)*	2.45 (1.82-3.29)***	2.05 (1.48-2.82)***
High * Poor	4.4(4.1-4.7)	0.56 (0.47-0.67)***	0.52 (0.43-0.62)***¤	0.53 (0.44-0.63)***	0.51 (0.42-0.62)***	0.98 (0.67-1.44)	0.70 (0.45-1.09)¤
High * Average	16.8(16.4-17.4)	0.44 (0.39-0.49)***	0.41 (0.37-0.47)***¤	0.42 (0.37-0.47)***	0.40 (0.35-0.45)***¤	0.78 (0.60-1.00)	0.70 (0.43-1.14)
High * Rich	6.9(6.6-7.3)	0.41 (0.35-0.49)***	0.40 (0.34-0.47)***	0.40 (0.34-0.48)***	0.40 (0.33-0.47)***	0.95 (0.69-1.32)	0.81 (0.45-1.48)
Don't know * Poor	5.1(4.8-5.4)	0.72 (0.62-0.83)***	0.71 (0.61-0.82)***	0.70 (0.60-0.81)***	0.70 (0.60-0.82)***	0.90 (0.64-1.27)	0.93 (0.68-1.27)
Don't know * Average	12.2(11.7-12.6)	0.68 (0.61-0.76)***	0.66 (0.59-0.74)***¤	0.68 (0.61-0.75)***	0.67 (0.60-0.75)***	1.05 (0.83-1.34)	1.00 (0.78-1.28)
Don't know * Rich	1.7(1.5-1.9)	0.60 (0.47-0.76)***	0.58 (0.45-0.75)***¤	0.52 (0.41-0.68)***	0.52 (0.40-0.68)***¤	1.79 (1.20-2.66)***	1.60 (0.99-2.58)

Low: Parental education as "Secondary and below"; High: Parental education as "Tertiary".

[a] adjusted for school clustering effect.

[b] adjusted for sex, age, grade, highest parental education, family affluence, harm perception regarding tobacco smoke exposure, current tobacco use and school clustering effect.

* $P < 0.05$, **$P < 0.01$, ***$P < 0.001$.

[†] Linear trend; [‡] Curvilinear trend.

[¤] Interaction with current tobacco use.

© 2024 Chen T. et al.
